# Variation in State-Level Eviction Moratorium Protections and Mental Health Among US Adults During the COVID-19 Pandemic

**DOI:** 10.1001/jamanetworkopen.2021.39585

**Published:** 2021-12-17

**Authors:** Kathryn M. Leifheit, Craig E. Pollack, Julia Raifman, Gabriel L. Schwartz, Robert D. Koehler, Jackie V. Rodriguez Bronico, Emily A. Benfer, Frederick J. Zimmerman, Sabriya L. Linton

**Affiliations:** 1 Department of Health Policy and Management, University of California, Los Angeles Fielding School of Public Health, Los Angeles; 2 Department of Health Policy and Management, Johns Hopkins School of Public Health, Baltimore, Maryland; 3 Department of Medicine, Johns Hopkins University School of Medicine, Baltimore, Maryland; 4 Johns Hopkins School of Nursing, Baltimore, Maryland; 5 Department of Health Law, Policy, and Management, Boston University School of Public Health, Boston, Massachusetts; 6 Philip R. Lee Institute for Health Policy Studies, University of California, San Francisco, San Francisco; 7 Columbia Law School, New York, New York; 8 Community Health Improvement Department, Cambridge Health Alliance, Cambridge, Massachusetts; 9 Wake Forest University School of Law, Winston-Salem, North Carolina; 10 Department of Mental Health, Johns Hopkins University School of Public Health, Baltimore, Maryland

## Abstract

**Importance:**

Although evictions have been associated with adverse mental health outcomes, it remains unclear which stages of the eviction process are associated with mental distress among renters. Variation in COVID-19 pandemic eviction protections across US states enables identification of intervention targets within the eviction process to improve renters’ mental health.

**Objective:**

To measure the association between the strength of eviction protections (ie, stages blocked by eviction moratoriums) and mental distress among renters during the COVID-19 pandemic.

**Design, Setting, and Participants:**

This cohort study used individual-level, nationally representative data from the Understanding Coronavirus in America Survey to measure associations between state eviction moratorium protections and mental distress. The sample of 2317 respondents included renters with annual household incomes less than $75 000 who reported a state of residence and completed surveys between March 10 and September 3, 2020, prior to the federal eviction moratorium order by the Centers for Disease Control and Prevention.

**Exposures:**

Time-varying strength of state moratorium protections as a categorical variable: none, weak (blocking court hearings, judgments, or enforcement without blocking notice or filing), or strong (blocking all stages of the eviction process beginning with notice and filing).

**Main Outcomes and Measures:**

Moderate to severe mental distress was measured using the 4-item Patient Health Questionnaire. Linear regression models were adjusted for time-varying state COVID-19 incidence and mortality, public health restrictions, and unemployment rates. Models included individual and time fixed effects as well as clustered standard errors.

**Results:**

The sample consisted of 2317 individuals (20 853 total observations) composed largely (1788 [78%] weighted) of middle-aged adults (25-64 years of age) and women (1538 [60%]); 640 respondents (23%) self-reported as Hispanic or Latinx, 314 respondents (20%) as non-Hispanic Black, and 1071 respondents (48%) as non-Hispanic White race and ethnicity. Relative to no state-level eviction moratorium protections, strong protections were associated with a 12.6% relative reduction (risk ratio, 0.87; 95% CI, 0.76-0.99) in the probability of mental distress, whereas weak protections were not associated with a statistically significant reduction (risk ratio, 0.96; 95% CI, 0.86-1.06).

**Conclusions and Relevance:**

This analysis of the Understanding Coronavirus in America Survey data found that strong eviction moratoriums were associated with protection against mental distress, suggesting that distress begins early in the eviction process with notice and filing. This finding is consistent with the idea that to reduce mental distress among renters, policy makers should focus on primary prevention of evictions.

## Key Points

**Question:**

Which eviction protections were associated with reduced mental distress among renters during the COVID-19 pandemic?

**Findings:**

In this cohort study assessing data from 2317 adult respondents of the Understanding Coronavirus in America Survey, moratoriums that blocked landlords from giving notice or filing for evictions were associated with a relative reduction in mental distress of approximately 13%, whereas protections that blocked only court hearings, judgments, and enforcement did not reduce distress significantly.

**Meaning:**

Findings indicate that only moratoriums that blocked eviction notice and filing were associated with reduced renter distress, suggesting that to protect renters’ mental health, interventions should aim to prevent evictions before filing.

## Teaser

This cohort study uses individual-level data from respondents of the Understanding Coronavirus in America Survey to assess associations between various levels of eviction protections and mental distress among renters during the COVID-19 pandemic.

## Introduction

Housing insecurity, a factor know to be associated with mental distress,^1^ has been widespread in the US. Following decades of increasing housing costs, the median household income in the US is no longer adequate to pay for basic needs.^2^ In 2019, 1 in 3 renter households earning less than $75 000 lived in unaffordable housing, putting more than 30% of their income toward rent and utilities.^3^ Stretched thin, renters face eviction with troubling frequency. Nationally, there are 6 eviction filings annually for every 100 renter households^4^; the rate is higher among female-headed households, families with children, and Black and Latinx people.^5,6,7^ During the last decade, a growing body of evidence has linked eviction and the threat of eviction to poor mental health outcomes, such as depressive symptoms, anxiety, and suicide.^8,9,10,11,12,13^

During the COVID-19 pandemic, mass reductions in wages and unprecedented job loss exacerbated eviction risk for millions of renter households,^14^ and inability to pay rent was strongly associated with suicidal ideation in a study conducted during the COVID-19 pandemic.^15^ Both US states and the federal government enacted eviction moratoriums without explicit attention to this issue: the primary goal was stemming transmission of COVID-19 due to eviction and homelessness. With renters accumulating large rental debts during the COVID-19 crisis,^16^ eviction moratoriums were often the only thing preventing tenants from immediate housing loss.^17^ Given the strong ties between housing insecurity and mental health, these moratoriums may also have served to protect renters’ mental health. Accordingly, 1 prior study found that living in a state with an eviction moratorium was associated with lower levels of mental distress among Black survey respondents, although the association did not hold for the overall sample.^18^

Protections provided by state eviction moratoriums varied in ways that may be meaningful for mental health outcomes. This variation offers a rare opportunity to disentangle the ways that different stages of the eviction process are associated with mental distress. Evictions are generally thought of as a 5-stage process, beginning with landlords giving tenants notice of eviction, followed by landlords filing for eviction, a court hearing, a court judgment and order of eviction, and, finally, enforcement of the order (ie, tenants are forcibly removed). States that instituted stronger moratoriums intervened at the earlier stages, blocking landlords’ ability to threaten eviction via notice or filing, which may be especially important for mental health.^8^ Landlords often use the threat of eviction, including filing a case, to recoup rental debt.^19^ This threat puts mental strain on families as they work to prevent eviction and maintain stable housing.^20^ Yet many state eviction moratoriums blocked only the latter stages of eviction (ie, hearings, judgments, and enforcement), allowing landlords to provide notice of eviction and file cases during the pandemic.

In this cohort study, we sought to delineate whether different stages of the eviction process were associated with mental distress prior to the initiation of the federal eviction moratorium in a nationally representative sample of lower-income US renters. Specifically, we hypothesized that moratoriums on eviction notice and filing (ie, strong moratoriums) would be associated with reduced renter mental distress, whereas moratoriums that only intervened on blocked hearings, judgments, and enforcement (ie, weak moratoriums) would not. In secondary analyses, we explored whether the strength of these associations between stages of the eviction process and mental health varied according to affordability of the underlying housing market and across racial and ethnic groups.

## Methods

### Source and Study Population

We analyzed data from the Understanding Coronavirus in America Survey (UAS),^21^ a nationally representative, online panel survey conducted from March 10, 2020, and continuing through the COVID-19 crisis. The UAS is fielded in waves approximately every 2 weeks. This study adheres to the Strengthening the Reporting of Observational Studies in Epidemiology (STROBE) reporting guidelines for cohort studies. Because the data analyzed for this study are publicly available and are deidentified, this study did not meet the definition of human subjects research and was thus exempt from review by an institutional review board and did not require obtaining a waiver of consent from participants.

The UAS panel includes 8277 unique individuals, with response rates ranging from 67% to 84% across waves. We included respondents who lived in rental housing at any wave of the study (39% of the total sample) who had a baseline income below $75 000 per year (ie, annual household income in the past 12 months upon first survey; 76% of renters) and who completed surveys up to and including September 3, 2020, before a nationwide Centers for Disease Control and Prevention eviction moratorium became effective on September 4, 2020 (ie, UAS waves 1-13; 94% of renters with income <$75 000). We chose this income threshold for consistency with previous publications using the UAS data to evaluate the association of health with social policies meant to protect low-income and middle-class families.^22^ An additional 19 individuals were excluded owing to missing state of residence.

### Outcome

Our outcome of moderate or severe mental distress was measured using the 4-item Patient Health Questionnaire (PHQ4), a brief, validated^23,24^ questionnaire that measures levels of mental distress in the past 14 days on a scale ranging from 0 to 12. Scores greater than or equal to 6 indicate moderate or severe mental distress.

### Exposure

We classified time-varying moratorium strength using a data set collected and validated by the COVID-19 Eviction Moratoria & Housing Policy surveillance team.^25^ Trained housing lawyers, legal professionals, and student researchers (including R.D.K. and E.A.B.) conducted policy surveillance to describe state-level responses to the eviction crisis, including their duration and their substantive effect on the eviction process. We used the start and end dates of moratoria to create a time-varying indicator of whether individuals lived in a state with no protection, weak protection (ie, a moratorium blocking only hearings, judgments, or enforcement), or strong protection (a moratorium blocking notice or filing). We lagged the exposure by 14 days to measure the strength of moratorium protections at the beginning of the recall period for the PHQ4.

### Controls

We controlled for the following time-varying, state-level variables, all lagged by 14 days to align with the reporting period of the outcome: COVID-19 incidence and mortality (total confirmed cases and deaths per 100 000 population in the last 14 days, drawn from the Johns Hopkins Center for Systems Science and Engineering COVID-19 time series data^26^), monthly state unemployment rate per the US Bureau of Labor Statistics,^27^ and public health restrictions (binary indicators of shelter-in-place orders and school closures derived from the COVID-19 US state policy database^28^). Models included fixed effects for individuals to control for time-invariant characteristics of respondents (eg, age at baseline, gender, and race and ethnicity). We also included fixed effects for survey wave to control for underlying time trends and federal policies, such as the Coronavirus Aid, Relief, and Economic Security Act, which banned evictions from federally financed properties from March 27 through July 24, 2020.

### Statistical Analysis

#### Primary Analysis

We first describe moratorium protections over time and baseline characteristics of the study population, applying sample weights to estimate prevalence in the target population.

In our primary analysis, we measured the association between moratorium strength and mental distress using linear regression with individual fixed effects. We opted for linear regression over logistic regression given evidence that linear models produce less biased estimates in fixed-effects models with binary outcomes.^29^ Standard errors were clustered to account for correlation within individuals using the Huber-White sandwich estimator. Our statistical model is detailed in the following equation: *Y_ist_* = β_0_ + β_1_(Weak Moratorium)*_st_* _− 14_ + β_2_(Strong Moratorium)*_st_* _− 14_ + β_3_{[∑ *^t^_t_* _− 14_(Cases)*_st_*]/(Population)*_s_*} + β_4_{[∑ *^t^_t_* _− 14_(Deaths)*_st_*]/(Population)*_s_*} + β_5_(Unemployment)*_st_* _− 14_ + β_6_(Shelter in Place)*_st_* _− 14_ + β_7_(Schools Closed)*_st_* _− 14_ + μ*_i_* + θ*_t_* + ε*_ist_*, where *i* indexes the individual, *s* indexes the individual’s state of residence, and *t* indexes the survey date. Individual fixed effects are denoted with *μ_i_*, and time fixed effects are denoted with *θ_t_*. After regression, we estimated the adjusted prevalence of moderate and severe mental distress within categories of moratorium strength, with covariates set to mean values.

#### Secondary Analyses

We expected that eviction moratoriums might have stronger effects on mental health in states with less affordable rental housing and, consequently, greater eviction risk during the pandemic. To evaluate this possibility, we stratified respondents by state-level housing cost burden (greater than or less than the median state level of housing cost burden in 2019 [ie, the percentage of renters in the state dedicating >30% of their household income to rent]^30^). Finally, because Black and Hispanic tenants are disproportionately targets of eviction,^7,31^ we hypothesized that there would be variation in moratorium effects by race and ethnicity. To test this hypothesis, we stratified by respondent self-reported race and ethnicity (Hispanic, non-Hispanic Black, and non-Hispanic White).

#### Sensitivity Analyses

We conducted a number of sensitivity analyses. First, to test the sensitivity of estimates to the income cutoff used for inclusion, we limited our study to renters with baseline household incomes below $50 000 per year rather than $75 000 as in the main analysis. Second, because moratorium status has been associated with subsequent COVID-19 incidence and mortality,^32^ COVID-19 incidence and mortality could potentially mediate, and therefore attenuate, the association between moratorium strength and mental health. To guard against this outcome, we lagged COVID-19 incidence and mortality controls by an additional week such that COVID-19 variables would precede policy decisions regarding moratorium strength. Third, renters’ mental distress could increase in anticipation of moratorium expiration. To explore potential associations with anticipation, we lagged the exposure by only 1 week such that the 2-week recall period for the PHQ4 included time leading up to moratorium expiration. Fourth, the main regression models were unweighted because unweighted ordinary least-squares estimates of associations may be more precise and less biased than weighted estimates.^33,34^ In sensitivity analyses, we conducted regressions incorporating the UAS weights. Fifth, because our exposure occurred at the state level, there is an argument to be made for clustering standard errors within states rather than individuals. To test whether our results were sensitive to the level of clustering specified, we conducted 2 regressions clustered by state, with 1 analysis excluding states with sparse sampling (<40 individuals). Sixth, we modeled associations including additional covariates for household-level, time-varying risk factors for mental distress (death or hospitalization of a close friend of family member due to COVID-19, unemployment, reduction in work hours, or receipt of stimulus funds). These household-level factors were not included in the main models because they are unlikely to cause state-level eviction policy and thus do not meet the standard for confounding control. Seventh, we varied our outcome definition to measure effects of moratorium strength on mild or greater distress (PHQ4 ≥ 3), severe mental distress (PHQ4 ≥ 9), and mean PHQ4 score. All analyses were conducted in Stata/SE, version 15.1 (StataCorp LLC).

## Results

### Description of the Study Population

The study population included 2317 individuals with 20 853 observations (mean [SD], 9 [3] observations per individual). The study population was composed largely (1788 [78%] weighted) of middle-aged adults (25-64 years of age), and 1538 respondents (60%) were women, whereas 779 (40%) were men (Table). Of 2317 respondents, 640 (23%) were Hispanic or Latinx, 314 (20%) were non-Hispanic Black, and 1071 (48%) were non-Hispanic White race and ethnicity. A total of 572 respondents (18%) held a Bachelor’s degree, 971 respondents (45%) reported a household income below $25 000 per year, 1611 respondents (67%) lived in households without children, and 1675 (64%) lived in a state with high housing cost burden.

**Table.** Baseline Characteristics of 2317 Lower-Income Renters in the Understanding Coronavirus in America Survey, March 10 to September 3, 2020

Characteristic	Renters, No. (%)^a^
Age group, y	
18-24	223 (10)
25-44	1064 (49)
45-64	724 (29)
≥65	306 (12)
Gender	
Female	1538 (60)
Male	779 (40)
Self-reported race and ethnicity	
Hispanic or Latinx	640 (23)
Non-Hispanic American Indian or Alaska Native	30 (<1)
Non-Hispanic Asian	137 (5)
Non-Hispanic Black	314 (20)
Non-Hispanic Hawaiian or Other Pacific Islander	12 (<1)
Non-Hispanic Mixed Race	109 (4)
Non-Hispanic White	1071 (48)
Missing	4 (<1)
Highest level of education	
<High school	221 (14)
High school diploma	531 (37)
Some college or technical school	993 (31)
Bachelor’s degree or higher	572 (18)
Annual household income, $	
<25 000	971 (45)
25 000 to <50 000	779 (33)
50 000 to 75 000	576 (22)
Household structure	
1 Adult, 0 children	618 (24)
≥2 Adults, 0 children	993 (43)
≥2 Adults, ≥1 child	596 (28)
1 Adult, ≥1 children	110 (5)
State housing cost burden^b^	
Low (<2019 median value of 42.3%)	642 (37)
High (≥2019 median value of 42.3%)	1675 (64)

^a^ Percentages incorporate sampling weights.

^b^ Percentage of households paying greater than 30% of income for rent in the 2019 American Community Survey.

**Figure 1.**
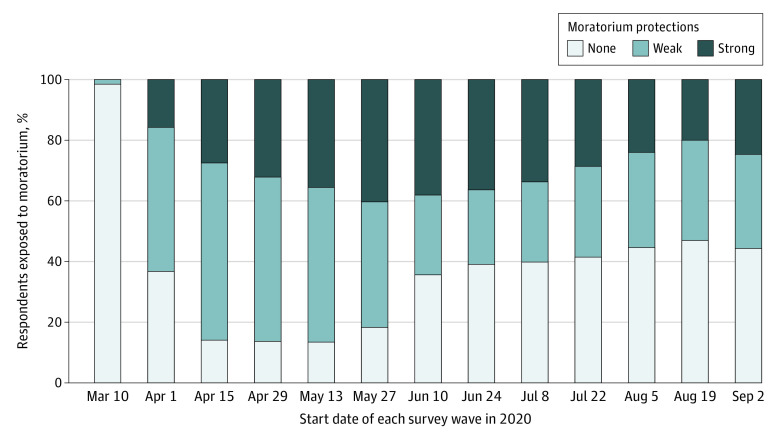
Eviction Protections by Survey Wave Among 2317 Participants in the Understanding Coronavirus in America Survey, March 10 to September 3, 2020 Horizontal axis labels indicate the first day of data collection for each survey wave. Percentages of exposed individuals on the vertical axis incorporate sampling weights.

Figure 1 shows the sample’s exposure to state-level moratoriums by week. State moratoriums were most often implemented in late March 2020 between UAS waves 1 and 2. Rates of moratorium protection (either strong or weak) were highest in survey waves 3 to 6, corresponding to April 15 to June 9, 2020, and tapered off thereafter.

### Primary Analysis

Results from the fixed-effects linear probability model suggest that residing in a state with a weak eviction moratorium was associated with a nonstatistically significant 0.7 percentage-point reduction (95% CI, −1.1 to 2.6 percentage points) in the probability of moderate to severe mental distress, relative to living in a state with no eviction moratorium. However, the estimated reduction comparing individuals in states with strong moratoriums to those in states without moratoriums was statistically significant at 2.2 percentage points (95% CI, 0.03 to 4.5 percentage points). Full regression model results are provided in the eTable in the Supplement.

**Figure 2.**
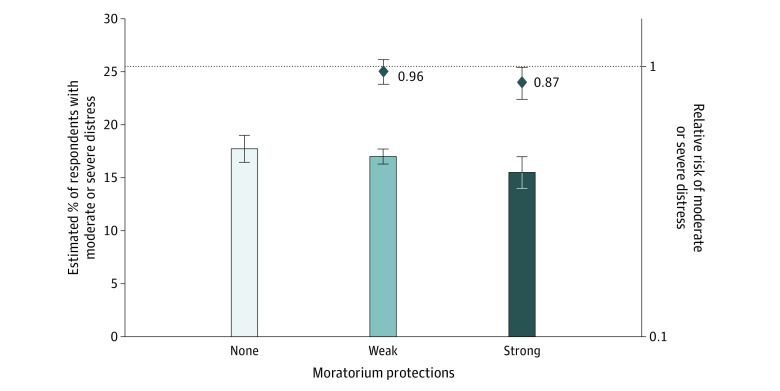
Association of Eviction Moratorium Strength With Moderate or Severe Mental Distress Among 2317 US Renters Responding to the Understanding Coronavirus in America Survey, March 10 to September 3, 2020 Bars display the estimated prevalence of moderate or severe mental distress associated with each type of moratorium protection. Relative risk estimates compare prevalence in US states with weak and strong moratoriums to prevalence in US states with no moratorium. Estimates are adjusted for state COVID-19 incidence and mortality, unemployment rate, stay-at-home orders, and school closures in the last 14 days and include individual and time fixed effects and clustered standard errors.

The estimated prevalence of moderate to severe mental distress was 17.7% (95% CI, 16.5%-19.0%) (Figure 2) among individuals in states with no moratorium protection, 17.0% (95% CI, 16.3%-17.7%) in states with weak moratoriums, and 15.5% (95% CI, 14.0%-17.0%) in states with strong moratoriums. On a relative scale, therefore, weak moratoriums were associated with a 4.1% reduction in the probability of mental distress (risk ratio, 0.96; 95% CI, 0.86-1.06) and strong moratoriums were associated with a 12.6% reduction (risk ratio, 0.87; 95% CI, 0.76-0.99).

### Secondary Analyses

**Figure 3.**
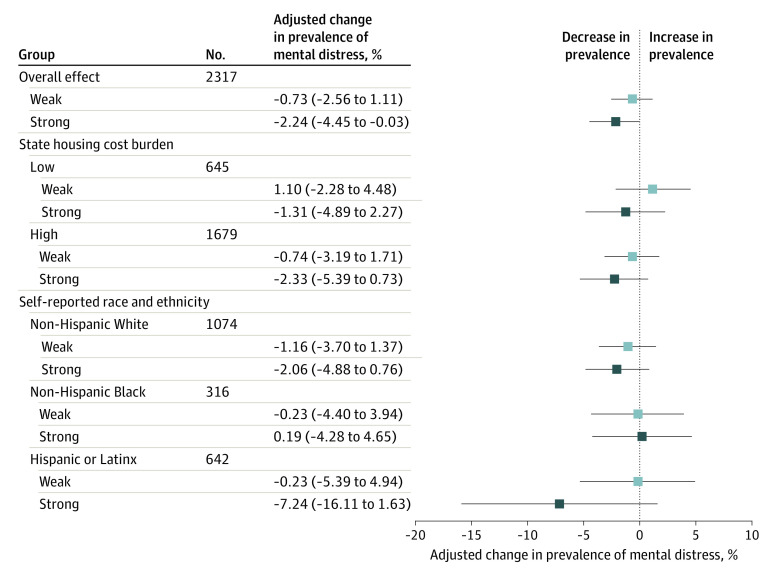
Results of Secondary Analyses, Stratifying Adjusted Associations Between Moratorium Strength and Mental Distress by Subgroups of State-Level Housing Cost Burden and Race and Ethnicity Adjusted absolute change in prevalence of moderate or severe mental distress, relative to no moratorium protection. Low cost burden is defined as a US state with fewer than 42.3% of renters dedicating more than 30% of their household income to rent; 42.3% is the median rate of renter cost burden among US states in the 2019 American Community Survey. Linear probability regression adjusted for state COVID-19 incidence and mortality, unemployment rate, stay-at-home orders, and school closures in the last 14 days, with individual and time fixed effects and clustered standard errors. Error bars indicate 95% CIs; light blue boxes, weak moratorium; and dark blue boxes, strong moratorium.

Although they were not statistically significant, stratified analyses revealed that strong protections were associated with larger reductions in mental distress in some housing-policy contexts and for specific racial and ethnic groups (Figure 3). When we stratified by status of rental cost burden before the COVID-19 pandemic, strong moratoriums were associated with larger reductions in mental distress in states with high rental cost burden at baseline than those with lower rental cost burden. When we stratified by racial and ethnic group, strong moratoriums were associated with larger reductions in mental distress among Hispanic and non-Hispanic White tenants than among non-Hispanic Black tenants. We detected no noteworthy reductions in mental distress associated with weak moratoriums in any subgroup.

### Sensitivity Analyses

Results were robust to a number of sensitivity analyses. Using analyses with an income cutoff of $50 000 for eligibility, incorporating an additional 1-week lag in controls for COVID-19 incidence and mortality, allowing for anticipation effects, incorporating survey weights, clustering by state, and incorporating additional household-level controls all produced findings consistent with the main analysis (eFigure in the Supplement). Although regressions measuring associations with severe distress and mean PHQ4 score produced qualitatively similar results to the main regression model, we found a null association between moratorium strength and mild or greater distress.

## Discussion

This cohort study analyzing UAS data found that strong moratoriums were associated with a significant reduction in mental distress during the COVID-19 pandemic, whereas weak moratoriums were not. This result is consistent with qualitative research showing that the stress of an eviction appears to begin early in the process, when housing insecurity and its legal ramifications first loom.^20^ We also found the association between strong moratoriums and better mental health to be particularly pronounced among individuals living in states with higher rental cost burden and for Hispanic and non-Hispanic White renters, suggesting care must be taken to ensure equitable benefit of these policies for mental health.

A 2 percentage-point absolute reduction in the prevalence of mental distress associated with strong eviction moratoriums equates to a 12.6% relative reduction in distress. In the context of nearly 33 million renter households in the US earning below $75 000, such protections could make a meaningful difference in the mental health of renters, many of whom are families with children. That we were able to detect a statistically significant association is meaningful, particularly given the context in which these policies were implemented. First, policy effects on mental health are likely dependent on renters’ knowledge and perceptions of protections offered by eviction moratoriums. The complex and changing status of moratorium protections might mean that renters had limited awareness of eviction moratoriums, in which case we would not expect large effects on mental distress. Evidence suggests that some number of evictions and forced moves proceeded in states with effective moratoriums, either owing to policy loopholes, illegal extrajudicial evictions, or incomplete enforcement.^35^ Knowledge of these policy violations might also have limited any protective associations of moratoriums with renters’ mental distress. Finally, it is important to acknowledge the context in which our study was conducted. The COVID-19 pandemic was marked with population-level increases in mental distress.^36,37^ Lower-income renters in our study were likely exposed to a range of stressors independent of eviction risk, including but not limited to social isolation, bereavement, heightened awareness of racialized police violence following the murder of George Floyd,^38,39^ and a tumultuous political climate leading up to the 2020 presidential election.^40^ These contemporaneous factors may limit the degree to which eviction moratoriums were associated with protection against mental distress.

We found that strong moratoriums were associated with larger reductions in mental distress in states with high rates of prepandemic renter cost burden, although these differences were not statistically significant. In addition, the association between strong moratoriums and mental distress was less pronounced for non-Hispanic Black renters than among Hispanic and non-Hispanic White renters, although again these findings did not reach statistical significance. Imprecision in these stratified analyses precludes us from making meaningful conclusions about effect heterogeneity. Future work is necessary to determine the potential for eviction policies to have varied effects in differing housing markets and to investigate whether seemingly race-neutral eviction policies have unequal benefits on both eviction prevention and other outcomes, including mental health.

### Limitations

Our analysis has a number of limitations. Sample size limited our statistical power in stratified analyses. In addition, because we studied eviction moratorium protections rather than actual eviction filings or enforcement, our results should be viewed as intention-to-treat effect estimates and may underestimate true associations between effective renter protections and mental distress. Although we controlled for time-invariant individual characteristics and time-varying features of states, the possibility of unmeasured, contemporaneous confounding remains. Finally, because UAS respondents are only identified by state of residence, we were unable to account for associations with city and county-level renter protections (eg, municipal eviction moratoriums or emergency rent relief).

Nonetheless, the results reported here provide evidence that intervening to prevent eviction notice and filings is associated with the prevention of mental distress among renters. Our results reinforce the ideas that the damage wrought by evictions begins early in the process and that the benefits of eviction prevention are immediate. An eviction notice or filing, regardless of whether it ends in a judgment against a tenant or a forced move, may bring about significant mental distress. In light of this evidence, policy makers should focus their efforts on primary prevention of evictions. In most states, landlords can file for evictions quickly, immediately after a late or partial rent payment, with little documentation and minimal fees.^41,42^ Many existing interventions to prevent evictions only become available to renters after their landlord files for eviction. Our findings suggest that, at least with respect to mental distress, these interventions may be too late. To reduce filings and prevent mental distress among low-income renters, states might consider increasing filing fees. Even more critical are policies and community-driven approaches that address the root causes of eviction by increasing availability of and access to affordable housing, establishing livable wages and meaningful safety nets, and eliminating racial discrimination in housing and economic markets.

## Conclusions

As the COVID-19 pandemic continues, eviction moratoriums are due to expire before rent relief reaches the tenants who need it. While tenants brace for a wave of evictions, our cohort study provides actionable evidence suggesting that eviction filings alone are associated with damage to renters’ mental health, and strong eviction protections may play an important role in promoting health equity.

## Notes

Supplement. **eTable.** Full Regression Results From the Linear Probability Model **eFigure.** Results of Sensitivity Analyses Modeling the Association Between Eviction Moratorium Strength and Moderate or Severe Mental Distress Among Renters in the Understanding Coronavirus in America Survey
