# Supplementary material for: Variation in State-Level Eviction Moratorium Protections and Mental Health Among US Adults During the COVID-19 Pandemic
Source: JAMA Netw Open. 2021 Dec 17;4(12):e2139585. doi: 10.1001/jamanetworkopen.2021.39585 (PMC8683968; doi:10.1001/jamanetworkopen.2021.39585)
Supplement: Supplement. — eTable. Full Regression Results From the Linear Probability Model eFigure. Results of Sensitivity Analyses Modeling the Association Between Eviction Moratorium Strength and Moderate or Severe Mental Distress Among Renters in the Understanding Coronavirus in America Survey [file jamanetwopen-e2139585-s001.pdf]

Supplementary Online Content

Leifheit KM, Pollack CE, Raifman J, et al. Variation in state-level eviction moratorium protections and mental health among US adults during the COVID-19 pandemic. *JAMA Netw Open*. 2021;4(12):e2139585. doi:10.1001/jamanetworkopen.2021.39585

eTable. Full Regression Results From the Linear Probability Model

eFigure. Results of Sensitivity Analyses Modeling the Association Between Eviction Moratorium Strength and Moderate or Severe Mental Distress Among Renters in the Understanding Coronavirus in America Survey

This supplementary material has been provided by the authors to give readers additional information about their work.

© 2021 Leifheit KM et al. *JAMA Network Open*.

eTable. Full Regression Results From the Linear Probability Model
(N=2317 individuals, 20853 observations)
Includes individual and time (survey wave) fixed effects.
Standard errors are adjusted for clustering at the individual level.

Independent variable	Coefficient	p-value	95% Confidence Interval
Moratorium protections			
None	[reference]		
Weak	-0.0073	0.437	-0.0256, 0.0111
Strong	-0.0224	0.047	-0.0445, -0.0003
Time-varying contextual variables (state-level)			
COVID-19 incidence (cases per 100,000)	0.0000183	0.598	-0.0000498, 0.0000864
COVID-19 mortality (deaths per 100,000)	-0.0001	0.823	-0.0016, 0.0012
Unemployment rate (%)	0.0018	0.254	-0.0013, 0.0050
Stay-at-home order in place	-0.0032	0.696	-0.0193, 0.0013
Schools closed	-0.0114	0.635	-0.0587, 0.0359

© 2021 Leifheit KM et al. *JAMA Network Open*.

eFigure. Results of Sensitivity Analyses Modeling the Association Between Eviction Moratorium Strength and Moderate or Severe Mental Distress Among Renters in the Understanding Coronavirus in America Survey

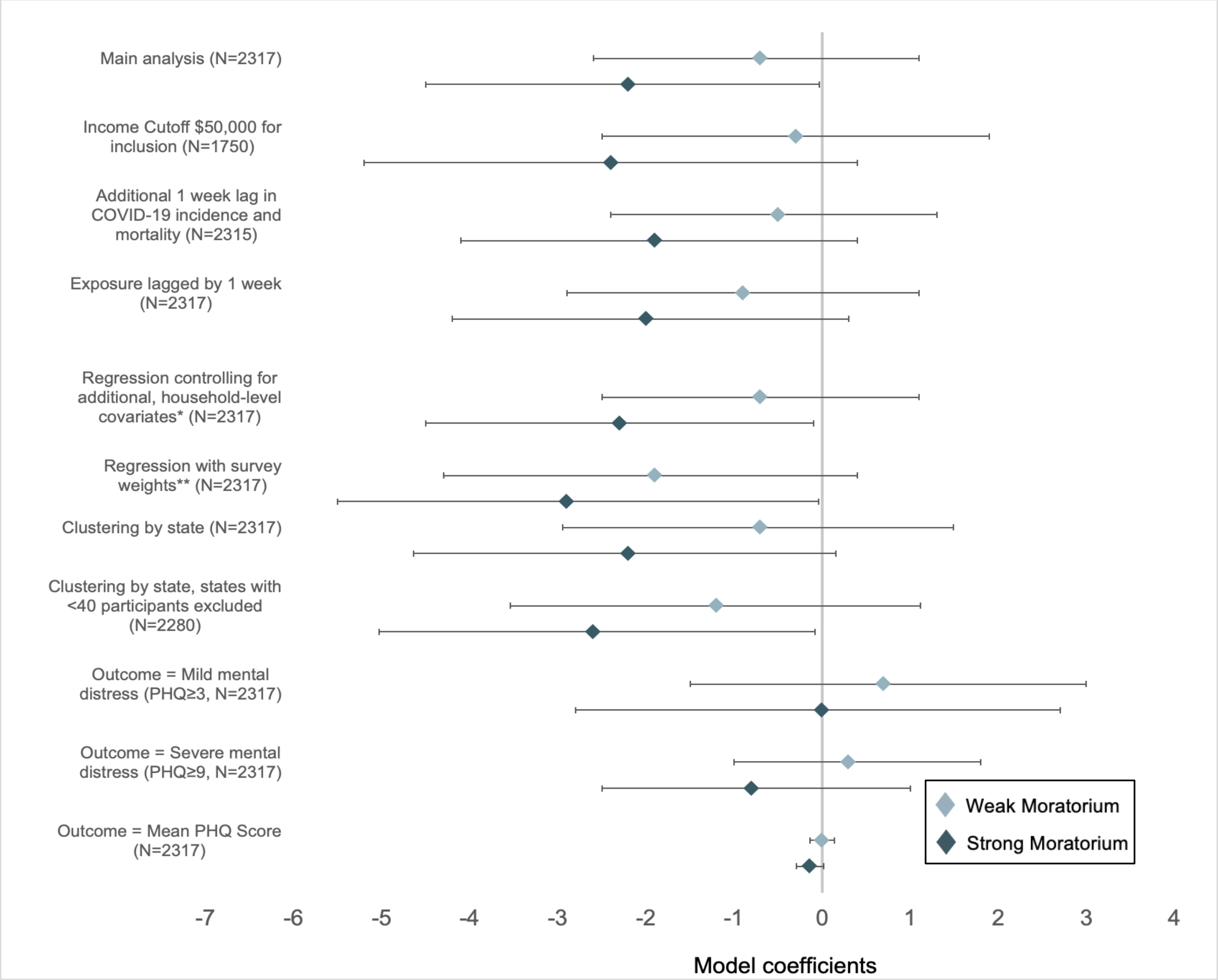

© 2021 Leifheit KM et al. *JAMA Network Open*.

All models include covariates for state COVID-19 incidence and mortality, unemployment rate, stay-at-home orders, and school closures in the past 14 days and include individual and time fixed effects and clustered standard errors. Coefficients for all but the final model can be interpreted as absolute differences in the predicted probability of distress associated with each type of moratorium protection. Coefficients for the final model can be interpreted as absolute differences in the mean PQH score (ranging from 0 to 12) associated with each type of moratorium protection.

*****Additional covariates included time varying dummy variables indicating death or hospitalization of a close friend of family member due to COVID-19, unemployment, reduction in work hours, receipt of stimulus funds.
**We apply the mean survey weight across observations.

© 2021 Leifheit KM et al. *JAMA Network Open*.
